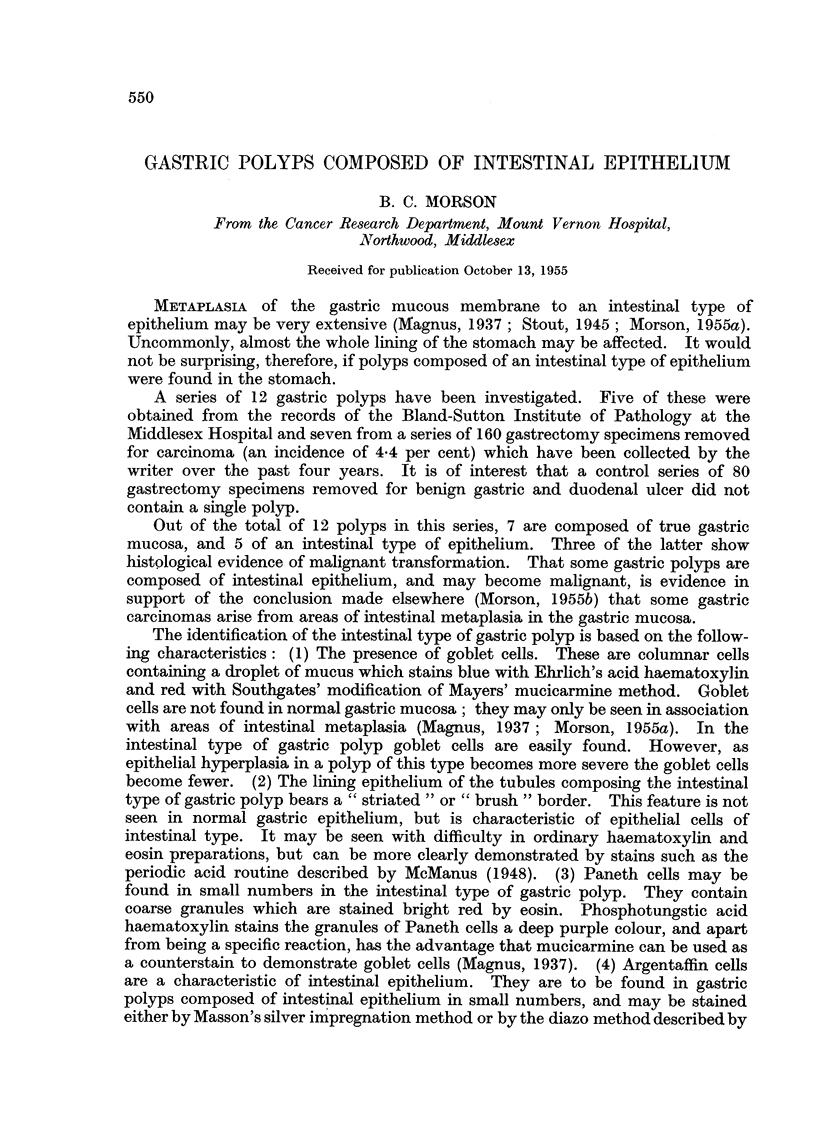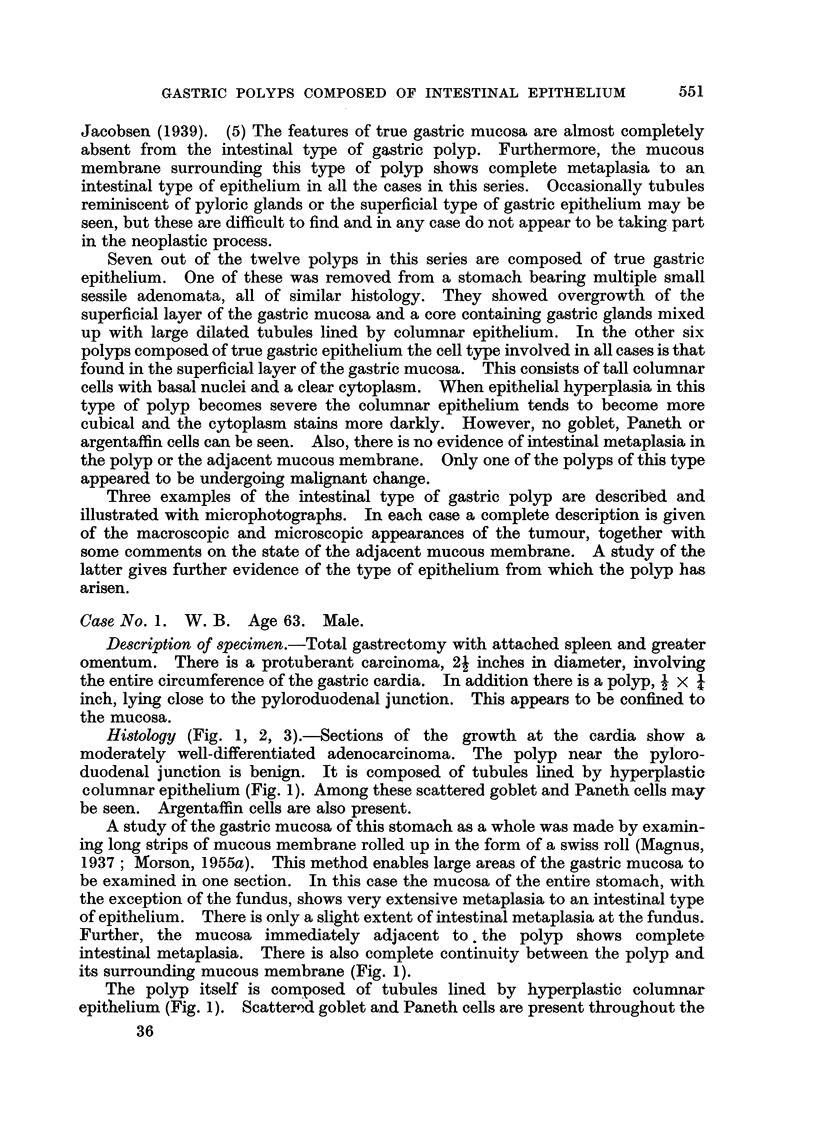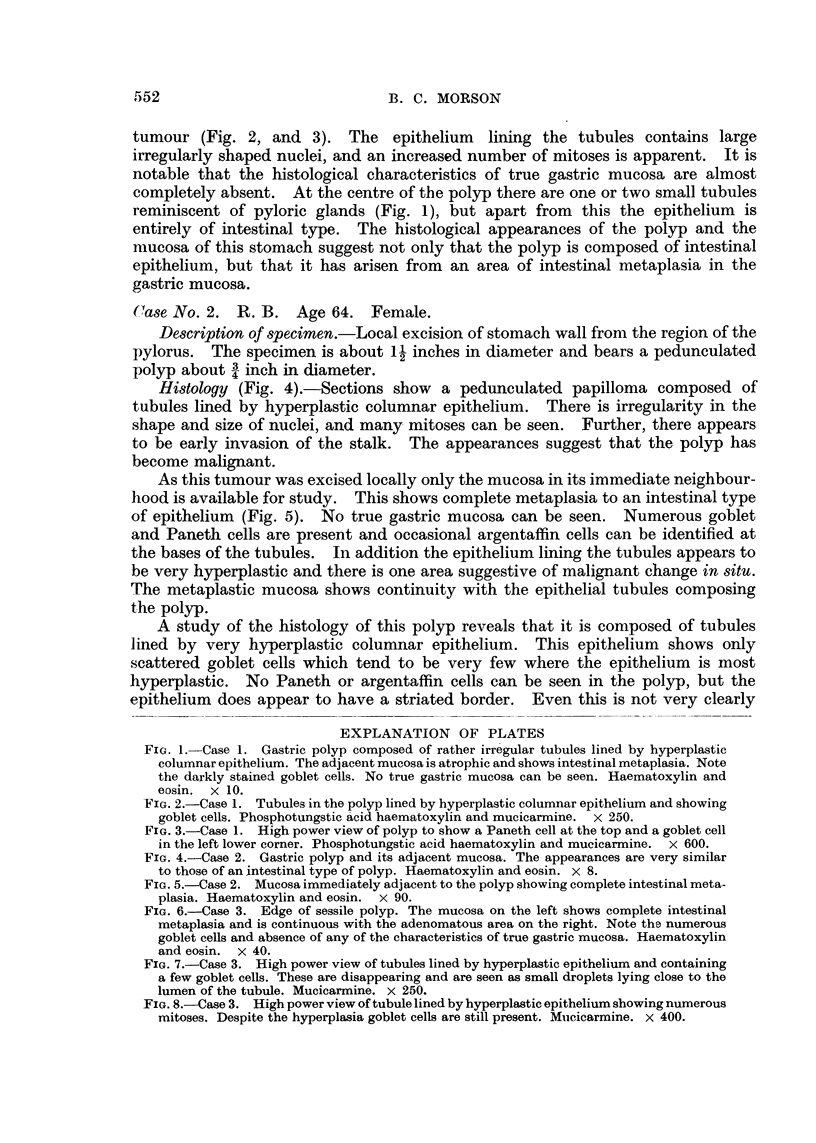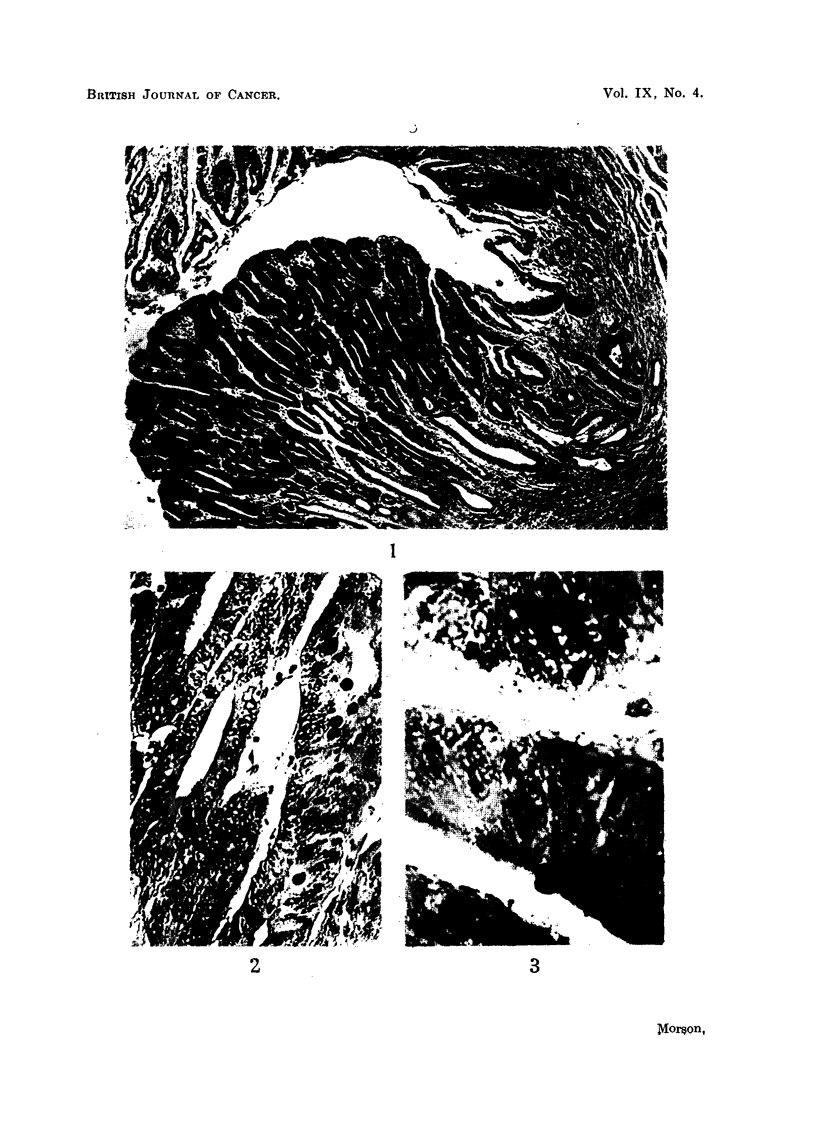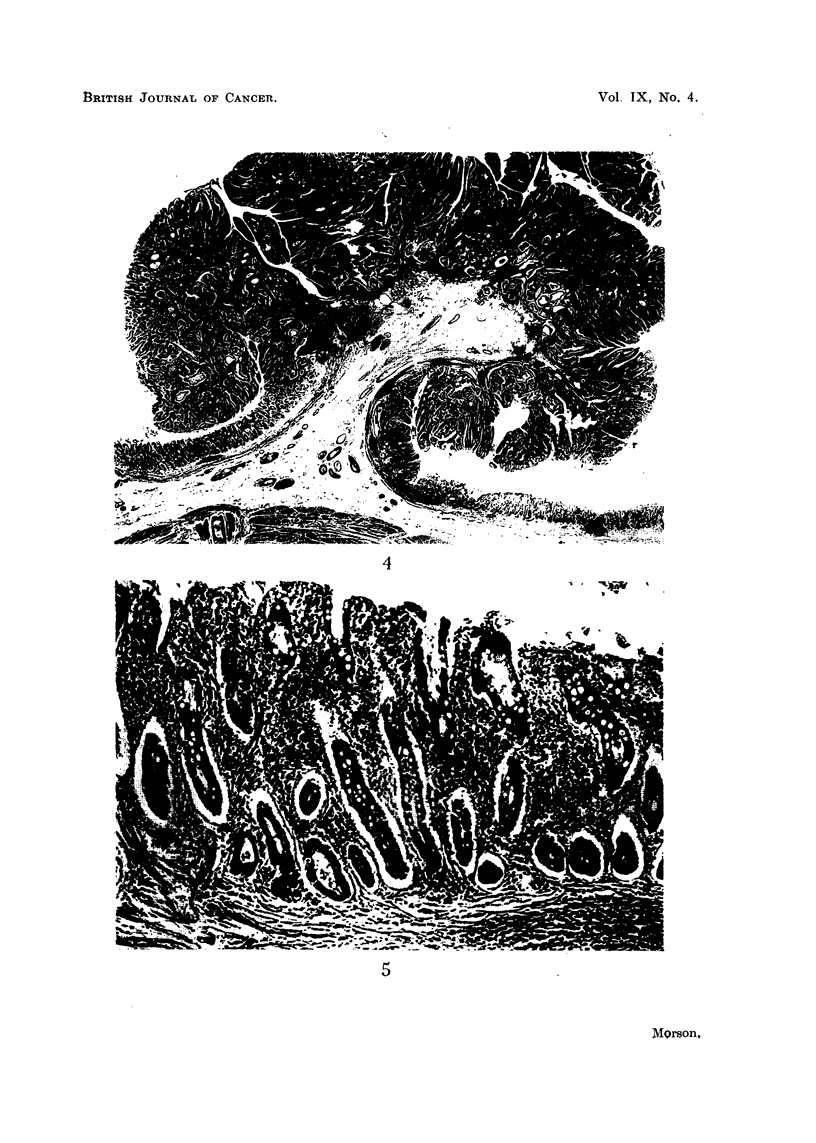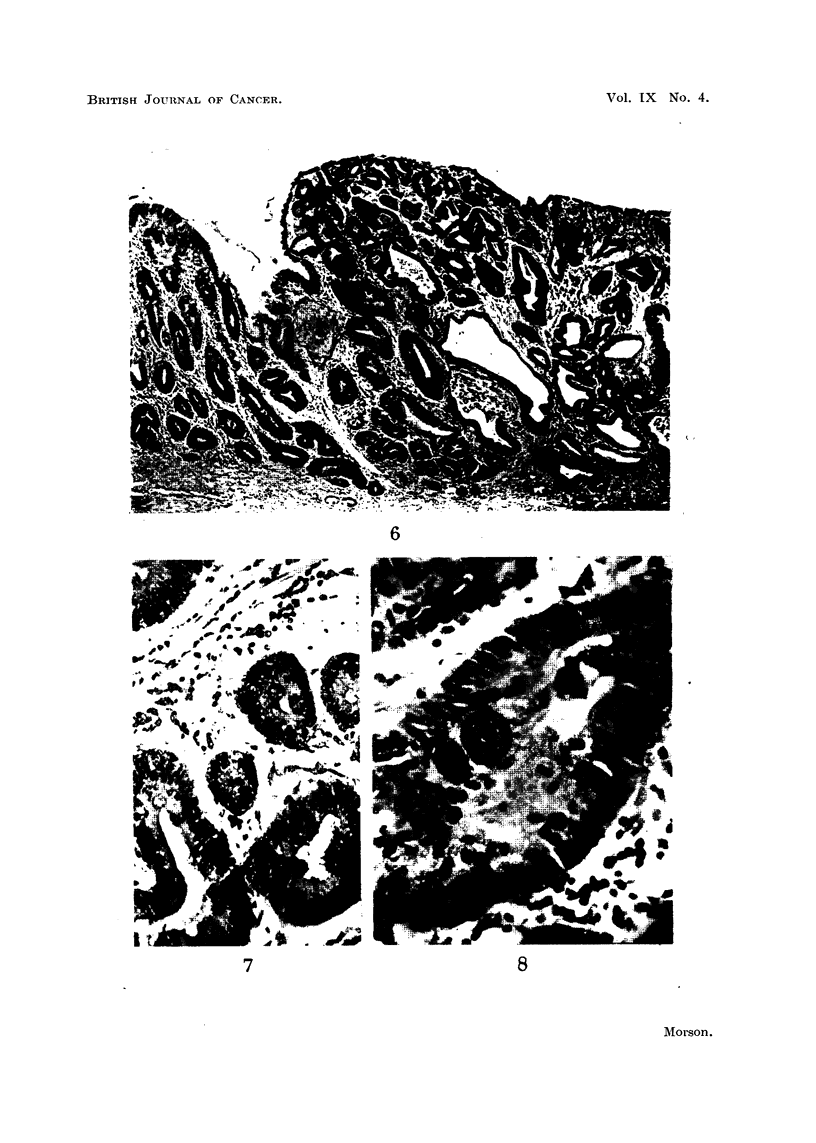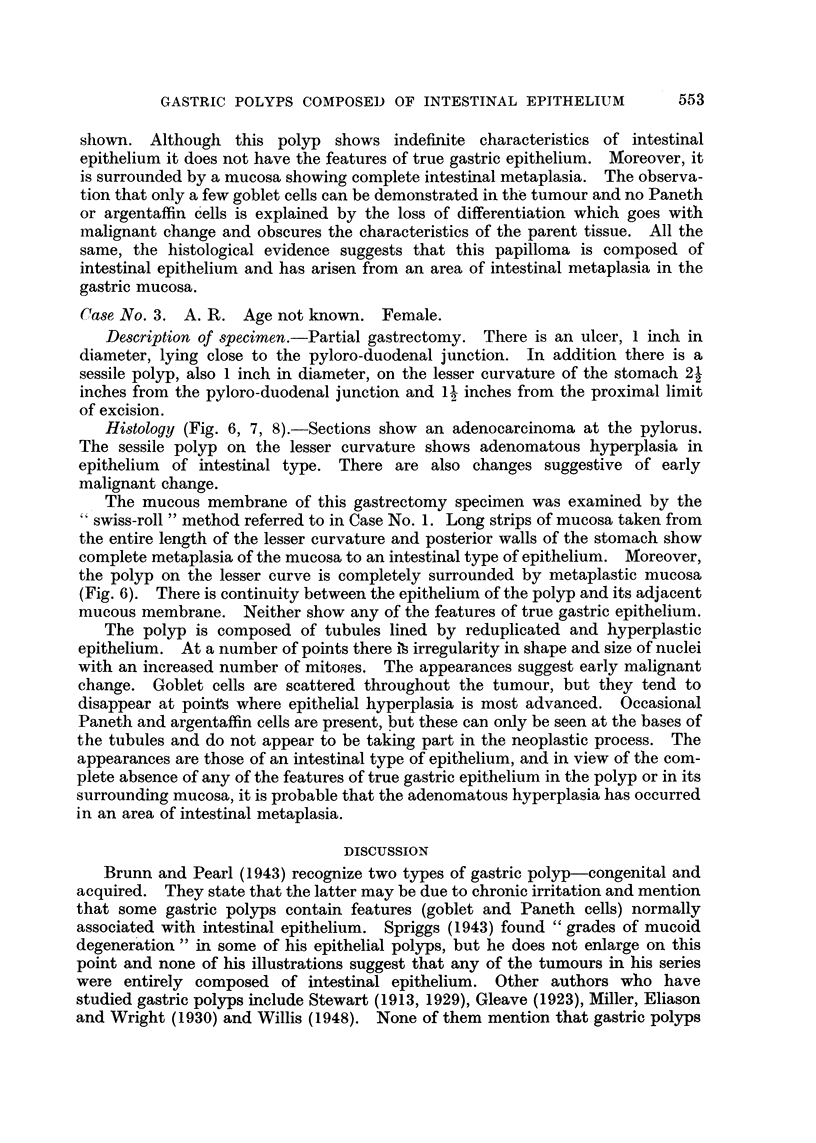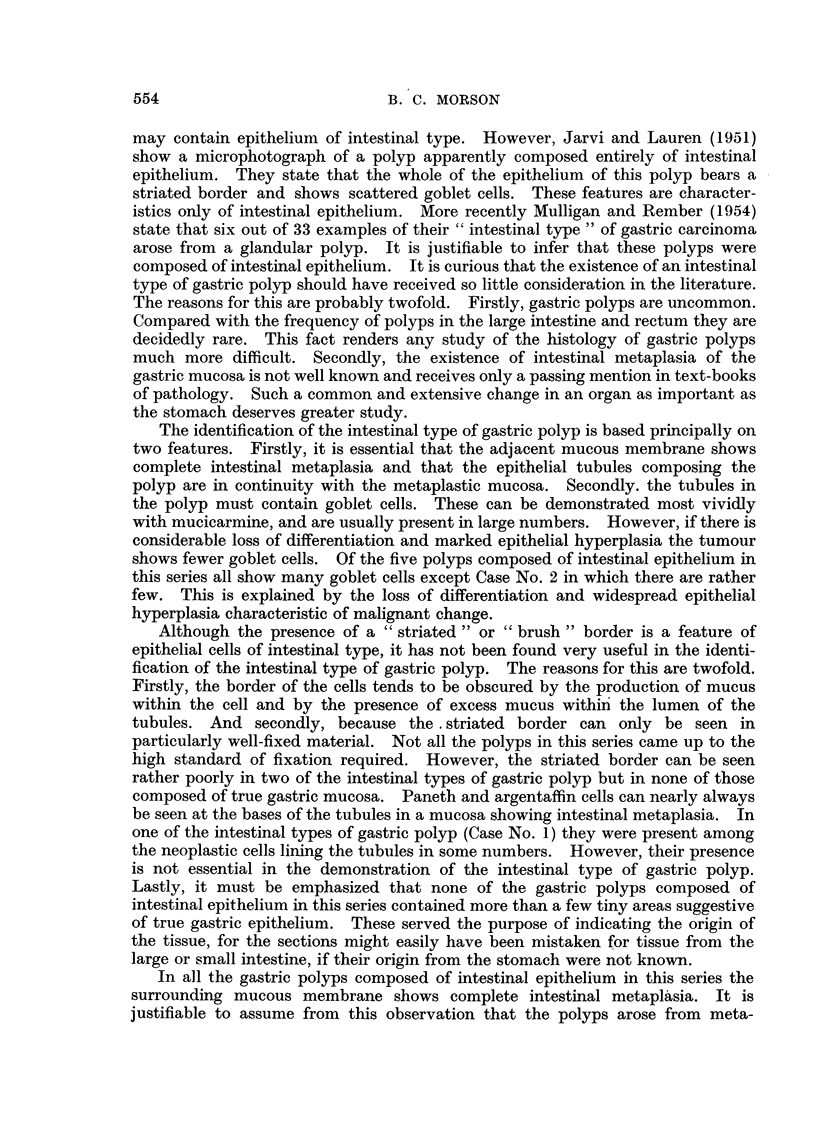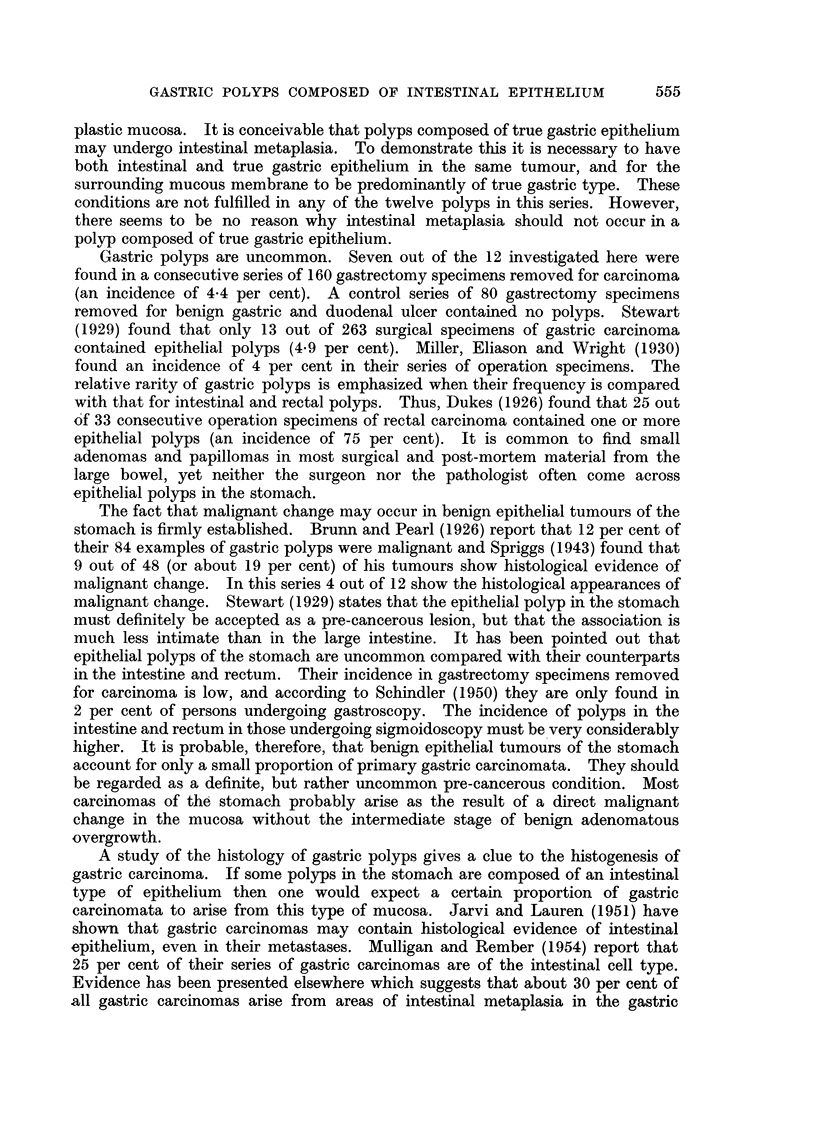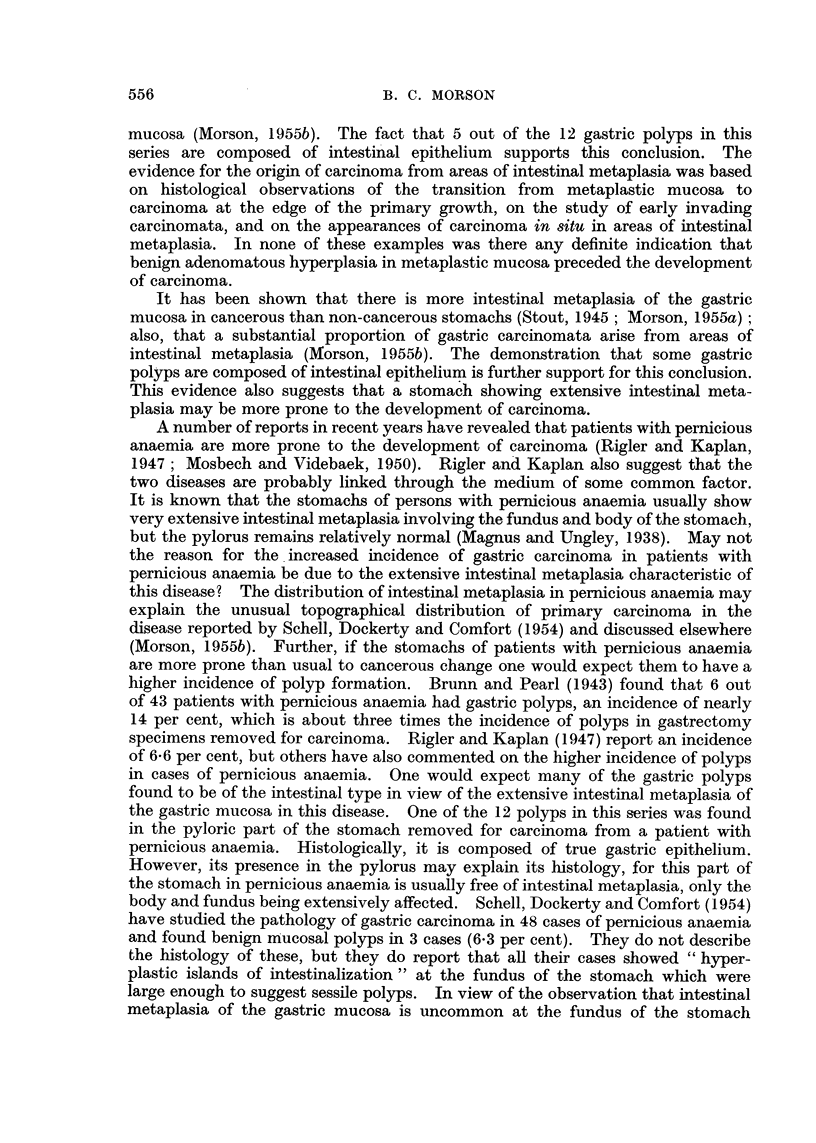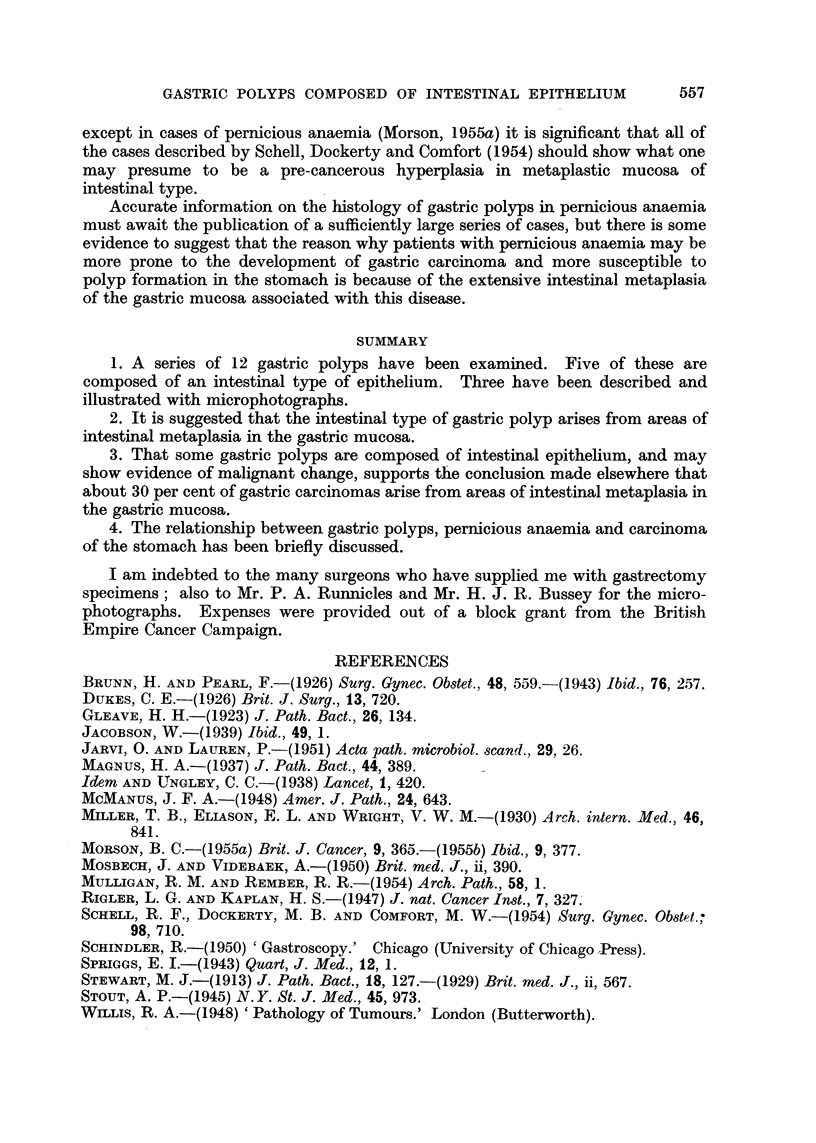# Gastric Polyps Composed of Intestinal Epithelium

**DOI:** 10.1038/bjc.1955.56

**Published:** 1955-12

**Authors:** B. C. Morson

## Abstract

**Images:**


### GASTRIC POLYPS COMPOSED OF INTESTINAL EPITHELIUM

B. C. MORSON

*From the Cancer Research Department, Mount Vernon Hospital, Northwood, Middlesex*

Received for publication October 13, 1955

METAPLASIA of the gastric mucous membrane to an intestinal type of epithelium may be very extensive (Magnus, 1937 ; Stout, 1945 ; Morson, 1955*a*). Uncommonly, almost the whole lining of the stomach may be affected. It would not be surprising, therefore, if polyps composed of an intestinal type of epithelium were found in the stomach.

A series of 12 gastric polyps have been investigated. Five of these were obtained from the records of the Bland-Sutton Institute of Pathology at the Middlesex Hospital and seven from a series of 160 gastrectomy specimens removed for carcinoma (an incidence of 4·4 per cent) which have been collected by the writer over the past four years. It is of interest that a control series of 80 gastrectomy specimens removed for benign gastric and duodenal ulcer did not contain a single polyp.

Out of the total of 12 polyps in this series, 7 are composed of true gastric mucosa, and 5 of an intestinal type of epithelium. Three of the latter show histological evidence of malignant transformation. That some gastric polyps are composed of intestinal epithelium, and may become malignant, is evidence in support of the conclusion made elsewhere (Morson, 1955*b*) that some gastric carcinomas arise from areas of intestinal metaplasia in the gastric mucosa.

The identification of the intestinal type of gastric polyp is based on the following characteristics : (1) The presence of goblet cells. These are columnar cells containing a droplet of mucus which stains blue with Ehrlich's acid haematoxylin and red with Southgates' modification of Mayers' mucicarmine method. Goblet cells are not found in normal gastric mucosa ; they may only be seen in association with areas of intestinal metaplasia (Magnus, 1937 ; Morson, 1955*a*). In the intestinal type of gastric polyp goblet cells are easily found. However, as epithelial hyperplasia in a polyp of this type becomes more severe the goblet cells become fewer. (2) The lining epithelium of the tubules composing the intestinal type of gastric polyp bears a " striated " or " brush " border. This feature is not seen in normal gastric epithelium, but is characteristic of epithelial cells of intestinal type. It may be seen with difficulty in ordinary haematoxylin and eosin preparations, but can be more clearly demonstrated by stains such as the periodic acid routine described by McManus (1948). (3) Paneth cells may be found in small numbers in the intestinal type of gastric polyp. They contain coarse granules which are stained bright red by eosin. Phosphotungstic acid haematoxylin stains the granules of Paneth cells a deep purple colour, and apart from being a specific reaction, has the advantage that mucicarmine can be used as a counterstain to demonstrate goblet cells (Magnus, 1937). (4) Argentaffin cells are a characteristic of intestinal epithelium. They are to be found in gastric polyps composed of intestinal epithelium in small numbers, and may be stained either by Masson's silver impregnation method or by the diazo method described by

Jacobsen (1939). (5) The features of true gastric mucosa are almost completely absent from the intestinal type of gastric polyp. Furthermore, the mucous membrane surrounding this type of polyp shows complete metaplasia to an intestinal type of epithelium in all the cases in this series. Occasionally tubules reminiscent of pyloric glands or the superficial type of gastric epithelium may be seen, but these are difficult to find and in any case do not appear to be taking part in the neoplastic process.

Seven out of the twelve polyps in this series are composed of true gastric epithelium. One of these was removed from a stomach bearing multiple small sessile adenomata, all of similar histology. They showed overgrowth of the superficial layer of the gastric mucosa and a core containing gastric glands mixed up with large dilated tubules lined by columnar epithelium. In the other six polyps composed of true gastric epithelium the cell type involved in all cases is that found in the superficial layer of the gastric mucosa. This consists of tall columnar cells with basal nuclei and a clear cytoplasm. When epithelial hyperplasia in this type of polyp becomes severe the columnar epithelium tends to become more cubical and the cytoplasm stains more darkly. However, no goblet, Paneth or argentaffin cells can be seen. Also, there is no evidence of intestinal metaplasia in the polyp or the adjacent mucous membrane. Only one of the polyps of this type appeared to be undergoing malignant change.

Three examples of the intestinal type of gastric polyp are described and illustrated with microphotographs. In each case a complete description is given of the macroscopic and microscopic appearances of the tumour, together with some comments on the state of the adjacent mucous membrane. A study of the latter gives further evidence of the type of epithelium from which the polyp has arisen.

*Case No.* 1. W. B. Age 63. Male.

*Description of specimen.*—Total gastrectomy with attached spleen and greater omentum. There is a protuberant carcinoma, $2\frac{1}{2}$ inches in diameter, involving the entire circumference of the gastric cardia. In addition there is a polyp, $\frac{1}{2} \times \frac{1}{4}$ inch, lying close to the pyloroduodenal junction. This appears to be confined to the mucosa.

*Histology* (Fig. 1, 2, 3).—Sections of the growth at the cardia show a moderately well-differentiated adenocarcinoma. The polyp near the pyloroduodenal junction is benign. It is composed of tubules lined by hyperplastic columnar epithelium (Fig. 1). Among these scattered goblet and Paneth cells may be seen. Argentaffin cells are also present.

A study of the gastric mucosa of this stomach as a whole was made by examining long strips of mucous membrane rolled up in the form of a swiss roll (Magnus, 1937 ; Morson, 1955*a*). This method enables large areas of the gastric mucosa to be examined in one section. In this case the mucosa of the entire stomach, with the exception of the fundus, shows very extensive metaplasia to an intestinal type of epithelium. There is only a slight extent of intestinal metaplasia at the fundus. Further, the mucosa immediately adjacent to the polyp shows complete intestinal metaplasia. There is also complete continuity between the polyp and its surrounding mucous membrane (Fig. 1).

The polyp itself is composed of tubules lined by hyperplastic columnar epithelium (Fig. 1). Scattered goblet and Paneth cells are present throughout the tumour (Fig. 2, and 3). The epithelium lining the tubules contains large irregularly shaped nuclei, and an increased number of mitoses is apparent. It is notable that the histological characteristics of true gastric mucosa are almost completely absent. At the centre of the polyp there are one or two small tubules reminiscent of pyloric glands (Fig. 1), but apart from this the epithelium is entirely of intestinal type. The histological appearances of the polyp and the mucosa of this stomach suggest not only that the polyp is composed of intestinal epithelium, but that it has arisen from an area of intestinal metaplasia in the gastric mucosa.

*Case No.* 2. R. B. Age 64. Female.

*Description of specimen.*—Local excision of stomach wall from the region of the pylorus. The specimen is about $1\frac{1}{2}$ inches in diameter and bears a pedunculated polyp about $\frac{3}{4}$ inch in diameter.

*Histology* (Fig. 4).—Sections show a pedunculated papilloma composed of tubules lined by hyperplastic columnar epithelium. There is irregularity in the shape and size of nuclei, and many mitoses can be seen. Further, there appears to be early invasion of the stalk. The appearances suggest that the polyp has become malignant.

As this tumour was excised locally only the mucosa in its immediate neighbourhood is available for study. This shows complete metaplasia to an intestinal type of epithelium (Fig. 5). No true gastric mucosa can be seen. Numerous goblet and Paneth cells are present and occasional argentaffin cells can be identified at the bases of the tubules. In addition the epithelium lining the tubules appears to be very hyperplastic and there is one area suggestive of malignant change *in situ.* The metaplastic mucosa shows continuity with the epithelial tubules composing the polyp.

A study of the histology of this polyp reveals that it is composed of tubules lined by very hyperplastic columnar epithelium. This epithelium shows only scattered goblet cells which tend to be very few where the epithelium is most hyperplastic. No Paneth or argentaffin cells can be seen in the polyp, but the epithelium does appear to have a striated border. Even this is not very clearly

---

EXPLANATION OF PLATES

FIG. 1.—Case 1. Gastric polyp composed of rather irregular tubules lined by hyperplastic columnar epithelium. The adjacent mucosa is atrophic and shows intestinal metaplasia. Note the darkly stained goblet cells. No true gastric mucosa can be seen. Haematoxylin and eosin. × 10.

FIG. 2.—Case 1. Tubules in the polyp lined by hyperplastic columnar epithelium and showing goblet cells. Phosphotungstic acid haematoxylin and mucicarmine. × 250.

FIG. 3.—Case 1. High power view of polyp to show a Paneth cell at the top and a goblet cell in the left lower corner. Phosphotungstic acid haematoxylin and mucicarmine. × 600.

FIG. 4.—Case 2. Gastric polyp and its adjacent mucosa. The appearances are very similar to those of an intestinal type of polyp. Haematoxylin and eosin. × 8.

FIG. 5.—Case 2. Mucosa immediately adjacent to the polyp showing complete intestinal metaplasia. Haematoxylin and eosin. × 90.

FIG. 6.—Case 3. Edge of sessile polyp. The mucosa on the left shows complete intestinal metaplasia and is continuous with the adenomatous area on the right. Note the numerous goblet cells and absence of any of the characteristics of true gastric mucosa. Haematoxylin and eosin. × 40.

FIG. 7.—Case 3. High power view of tubules lined by hyperplastic epithelium and containing a few goblet cells. These are disappearing and are seen as small droplets lying close to the lumen of the tubule. Mucicarmine. × 250.

FIG. 8.—Case 3. High power view of tubule lined by hyperplastic epithelium showing numerous mitoses. Despite the hyperplasia goblet cells are still present. Mucicarmine. × 400.

1

2

3

Morson.

4

5

6

7

8

shown. Although this polyp shows indefinite characteristics of intestinal epithelium it does not have the features of true gastric epithelium. Moreover, it is surrounded by a mucosa showing complete intestinal metaplasia. The observation that only a few goblet cells can be demonstrated in the tumour and no Paneth or argentaffin cells is explained by the loss of differentiation which goes with malignant change and obscures the characteristics of the parent tissue. All the same, the histological evidence suggests that this papilloma is composed of intestinal epithelium and has arisen from an area of intestinal metaplasia in the gastric mucosa.

*Case No.* 3. A. R. Age not known. Female.

*Description of specimen.*—Partial gastrectomy. There is an ulcer, 1 inch in diameter, lying close to the pyloro-duodenal junction. In addition there is a sessile polyp, also 1 inch in diameter, on the lesser curvature of the stomach $2\frac{1}{2}$ inches from the pyloro-duodenal junction and $1\frac{1}{2}$ inches from the proximal limit of excision.

*Histology* (Fig. 6, 7, 8).—Sections show an adenocarcinoma at the pylorus. The sessile polyp on the lesser curvature shows adenomatous hyperplasia in epithelium of intestinal type. There are also changes suggestive of early malignant change.

The mucous membrane of this gastrectomy specimen was examined by the " swiss-roll " method referred to in Case No. 1. Long strips of mucosa taken from the entire length of the lesser curvature and posterior walls of the stomach show complete metaplasia of the mucosa to an intestinal type of epithelium. Moreover, the polyp on the lesser curve is completely surrounded by metaplastic mucosa (Fig. 6). There is continuity between the epithelium of the polyp and its adjacent mucous membrane. Neither show any of the features of true gastric epithelium.

The polyp is composed of tubules lined by reduplicated and hyperplastic epithelium. At a number of points there is irregularity in shape and size of nuclei with an increased number of mitoses. The appearances suggest early malignant change. Goblet cells are scattered throughout the tumour, but they tend to disappear at points where epithelial hyperplasia is most advanced. Occasional Paneth and argentaffin cells are present, but these can only be seen at the bases of the tubules and do not appear to be taking part in the neoplastic process. The appearances are those of an intestinal type of epithelium, and in view of the complete absence of any of the features of true gastric epithelium in the polyp or in its surrounding mucosa, it is probable that the adenomatous hyperplasia has occurred in an area of intestinal metaplasia.

### DISCUSSION

Brunn and Pearl (1943) recognize two types of gastric polyp—congenital and acquired. They state that the latter may be due to chronic irritation and mention that some gastric polyps contain features (goblet and Paneth cells) normally associated with intestinal epithelium. Spriggs (1943) found " grades of mucoid degeneration " in some of his epithelial polyps, but he does not enlarge on this point and none of his illustrations suggest that any of the tumours in his series were entirely composed of intestinal epithelium. Other authors who have studied gastric polyps include Stewart (1913, 1929), Gleave (1923), Miller, Eliason and Wright (1930) and Willis (1948). None of them mention that gastric polyps may contain epithelium of intestinal type. However, Jarvi and Lauren (1951) show a microphotograph of a polyp apparently composed entirely of intestinal epithelium. They state that the whole of the epithelium of this polyp bears a striated border and shows scattered goblet cells. These features are characteristics only of intestinal epithelium. More recently Mulligan and Rember (1954) state that six out of 33 examples of their "intestinal type" of gastric carcinoma arose from a glandular polyp. It is justifiable to infer that these polyps were composed of intestinal epithelium. It is curious that the existence of an intestinal type of gastric polyp should have received so little consideration in the literature. The reasons for this are probably twofold. Firstly, gastric polyps are uncommon. Compared with the frequency of polyps in the large intestine and rectum they are decidedly rare. This fact renders any study of the histology of gastric polyps much more difficult. Secondly, the existence of intestinal metaplasia of the gastric mucosa is not well known and receives only a passing mention in text-books of pathology. Such a common and extensive change in an organ as important as the stomach deserves greater study.

The identification of the intestinal type of gastric polyp is based principally on two features. Firstly, it is essential that the adjacent mucous membrane shows complete intestinal metaplasia and that the epithelial tubules composing the polyp are in continuity with the metaplastic mucosa. Secondly. the tubules in the polyp must contain goblet cells. These can be demonstrated most vividly with mucicarmine, and are usually present in large numbers. However, if there is considerable loss of differentiation and marked epithelial hyperplasia the tumour shows fewer goblet cells. Of the five polyps composed of intestinal epithelium in this series all show many goblet cells except Case No. 2 in which there are rather few. This is explained by the loss of differentiation and widespread epithelial hyperplasia characteristic of malignant change.

Although the presence of a "striated" or "brush" border is a feature of epithelial cells of intestinal type, it has not been found very useful in the identification of the intestinal type of gastric polyp. The reasons for this are twofold. Firstly, the border of the cells tends to be obscured by the production of mucus within the cell and by the presence of excess mucus within the lumen of the tubules. And secondly, because the striated border can only be seen in particularly well-fixed material. Not all the polyps in this series came up to the high standard of fixation required. However, the striated border can be seen rather poorly in two of the intestinal types of gastric polyp but in none of those composed of true gastric mucosa. Paneth and argentaffin cells can nearly always be seen at the bases of the tubules in a mucosa showing intestinal metaplasia. In one of the intestinal types of gastric polyp (Case No. 1) they were present among the neoplastic cells lining the tubules in some numbers. However, their presence is not essential in the demonstration of the intestinal type of gastric polyp. Lastly, it must be emphasized that none of the gastric polyps composed of intestinal epithelium in this series contained more than a few tiny areas suggestive of true gastric epithelium. These served the purpose of indicating the origin of the tissue, for the sections might easily have been mistaken for tissue from the large or small intestine, if their origin from the stomach were not known.

In all the gastric polyps composed of intestinal epithelium in this series the surrounding mucous membrane shows complete intestinal metaplasia. It is justifiable to assume from this observation that the polyps arose from metaplastic mucosa. It is conceivable that polyps composed of true gastric epithelium may undergo intestinal metaplasia. To demonstrate this it is necessary to have both intestinal and true gastric epithelium in the same tumour, and for the surrounding mucous membrane to be predominantly of true gastric type. These conditions are not fulfilled in any of the twelve polyps in this series. However, there seems to be no reason why intestinal metaplasia should not occur in a polyp composed of true gastric epithelium.

Gastric polyps are uncommon. Seven out of the 12 investigated here were found in a consecutive series of 160 gastrectomy specimens removed for carcinoma (an incidence of 4·4 per cent). A control series of 80 gastrectomy specimens removed for benign gastric and duodenal ulcer contained no polyps. Stewart (1929) found that only 13 out of 263 surgical specimens of gastric carcinoma contained epithelial polyps (4·9 per cent). Miller, Eliason and Wright (1930) found an incidence of 4 per cent in their series of operation specimens. The relative rarity of gastric polyps is emphasized when their frequency is compared with that for intestinal and rectal polyps. Thus, Dukes (1926) found that 25 out of 33 consecutive operation specimens of rectal carcinoma contained one or more epithelial polyps (an incidence of 75 per cent). It is common to find small adenomas and papillomas in most surgical and post-mortem material from the large bowel, yet neither the surgeon nor the pathologist often come across epithelial polyps in the stomach.

The fact that malignant change may occur in benign epithelial tumours of the stomach is firmly established. Brunn and Pearl (1926) report that 12 per cent of their 84 examples of gastric polyps were malignant and Spriggs (1943) found that 9 out of 48 (or about 19 per cent) of his tumours show histological evidence of malignant change. In this series 4 out of 12 show the histological appearances of malignant change. Stewart (1929) states that the epithelial polyp in the stomach must definitely be accepted as a pre-cancerous lesion, but that the association is much less intimate than in the large intestine. It has been pointed out that epithelial polyps of the stomach are uncommon compared with their counterparts in the intestine and rectum. Their incidence in gastrectomy specimens removed for carcinoma is low, and according to Schindler (1950) they are only found in 2 per cent of persons undergoing gastroscopy. The incidence of polyps in the intestine and rectum in those undergoing sigmoidoscopy must be very considerably higher. It is probable, therefore, that benign epithelial tumours of the stomach account for only a small proportion of primary gastric carcinomata. They should be regarded as a definite, but rather uncommon pre-cancerous condition. Most carcinomas of the stomach probably arise as the result of a direct malignant change in the mucosa without the intermediate stage of benign adenomatous overgrowth.

A study of the histology of gastric polyps gives a clue to the histogenesis of gastric carcinoma. If some polyps in the stomach are composed of an intestinal type of epithelium then one would expect a certain proportion of gastric carcinomata to arise from this type of mucosa. Jarvi and Lauren (1951) have shown that gastric carcinomas may contain histological evidence of intestinal epithelium, even in their metastases. Mulligan and Rember (1954) report that 25 per cent of their series of gastric carcinomas are of the intestinal cell type. Evidence has been presented elsewhere which suggests that about 30 per cent of all gastric carcinomas arise from areas of intestinal metaplasia in the gastric mucosa (Morson, 1955*b*). The fact that 5 out of the 12 gastric polyps in this series are composed of intestinal epithelium supports this conclusion. The evidence for the origin of carcinoma from areas of intestinal metaplasia was based on histological observations of the transition from metaplastic mucosa to carcinoma at the edge of the primary growth, on the study of early invading carcinomata, and on the appearances of carcinoma *in situ* in areas of intestinal metaplasia. In none of these examples was there any definite indication that benign adenomatous hyperplasia in metaplastic mucosa preceded the development of carcinoma.

It has been shown that there is more intestinal metaplasia of the gastric mucosa in cancerous than non-cancerous stomachs (Stout, 1945 ; Morson, 1955*a*) ; also, that a substantial proportion of gastric carcinomata arise from areas of intestinal metaplasia (Morson, 1955*b*). The demonstration that some gastric polyps are composed of intestinal epithelium is further support for this conclusion. This evidence also suggests that a stomach showing extensive intestinal metaplasia may be more prone to the development of carcinoma.

A number of reports in recent years have revealed that patients with pernicious anaemia are more prone to the development of carcinoma (Rigler and Kaplan, 1947 ; Mosbech and Videbaek, 1950). Rigler and Kaplan also suggest that the two diseases are probably linked through the medium of some common factor. It is known that the stomachs of persons with pernicious anaemia usually show very extensive intestinal metaplasia involving the fundus and body of the stomach, but the pylorus remains relatively normal (Magnus and Ungley, 1938). May not the reason for the increased incidence of gastric carcinoma in patients with pernicious anaemia be due to the extensive intestinal metaplasia characteristic of this disease? The distribution of intestinal metaplasia in pernicious anaemia may explain the unusual topographical distribution of primary carcinoma in the disease reported by Schell, Dockerty and Comfort (1954) and discussed elsewhere (Morson, 1955*b*). Further, if the stomachs of patients with pernicious anaemia are more prone than usual to cancerous change one would expect them to have a higher incidence of polyp formation. Brunn and Pearl (1943) found that 6 out of 43 patients with pernicious anaemia had gastric polyps, an incidence of nearly 14 per cent, which is about three times the incidence of polyps in gastrectomy specimens removed for carcinoma. Rigler and Kaplan (1947) report an incidence of 6·6 per cent, but others have also commented on the higher incidence of polyps in cases of pernicious anaemia. One would expect many of the gastric polyps found to be of the intestinal type in view of the extensive intestinal metaplasia of the gastric mucosa in this disease. One of the 12 polyps in this series was found in the pyloric part of the stomach removed for carcinoma from a patient with pernicious anaemia. Histologically, it is composed of true gastric epithelium. However, its presence in the pylorus may explain its histology, for this part of the stomach in pernicious anaemia is usually free of intestinal metaplasia, only the body and fundus being extensively affected. Schell, Dockerty and Comfort (1954) have studied the pathology of gastric carcinoma in 48 cases of pernicious anaemia and found benign mucosal polyps in 3 cases (6·3 per cent). They do not describe the histology of these, but they do report that all their cases showed " hyperplastic islands of intestinalization " at the fundus of the stomach which were large enough to suggest sessile polyps. In view of the observation that intestinal metaplasia of the gastric mucosa is uncommon at the fundus of the stomach except in cases of pernicious anaemia (Morson, 1955*a*) it is significant that all of the cases described by Schell, Dockerty and Comfort (1954) should show what one may presume to be a pre-cancerous hyperplasia in metaplastic mucosa of intestinal type.

Accurate information on the histology of gastric polyps in pernicious anaemia must await the publication of a sufficiently large series of cases, but there is some evidence to suggest that the reason why patients with pernicious anaemia may be more prone to the development of gastric carcinoma and more susceptible to polyp formation in the stomach is because of the extensive intestinal metaplasia of the gastric mucosa associated with this disease.

### SUMMARY

1. A series of 12 gastric polyps have been examined. Five of these are composed of an intestinal type of epithelium. Three have been described and illustrated with microphotographs.
2. It is suggested that the intestinal type of gastric polyp arises from areas of intestinal metaplasia in the gastric mucosa.
3. That some gastric polyps are composed of intestinal epithelium, and may show evidence of malignant change, supports the conclusion made elsewhere that about 30 per cent of gastric carcinomas arise from areas of intestinal metaplasia in the gastric mucosa.
4. The relationship between gastric polyps, pernicious anaemia and carcinoma of the stomach has been briefly discussed.

I am indebted to the many surgeons who have supplied me with gastrectomy specimens ; also to Mr. P. A. Runnicles and Mr. H. J. R. Bussey for the microphotographs. Expenses were provided out of a block grant from the British Empire Cancer Campaign.

### REFERENCES

BRUNN, H. AND PEARL, F.—(1926) *Surg. Gynec. Obstet.*, **48**, 559.—(1943) *Ibid.*, **76**, 257.
DUKES, C. E.—(1926) *Brit. J. Surg.*, **13**, 720.
GLEAVE, H. H.—(1923) *J. Path. Bact.*, **26**, 134.
JACOBSON, W.—(1939) *Ibid.*, **49**, 1.
JARVI, O. AND LAUREN, P.—(1951) *Acta path. microbiol. scand.*, **29**, 26.
MAGNUS, H. A.—(1937) *J. Path. Bact.*, **44**, 389.
*Idem* AND UNGLEY, C. C.—(1938) *Lancet*, **1**, 420.
McMANUS, J. F. A.—(1948) *Amer. J. Path.*, **24**, 643.
MILLER, T. B., ELIASON, E. L. AND WRIGHT, V. W. M.—(1930) *Arch. intern. Med.*, **46**, 841.
MORSON, B. C.—(1955*a*) *Brit. J. Cancer*, **9**, 365.—(1955*b*) *Ibid.*, **9**, 377.
MOSBECH, J. AND VIDEBAEK, A.—(1950) *Brit. med. J.*, ii, 390.
MULLIGAN, R. M. AND REMBER, R. R.—(1954) *Arch. Path.*, **58**, 1.
RIGLER, L. G. AND KAPLAN, H. S.—(1947) *J. nat. Cancer Inst.*, **7**, 327.
SCHELL, R. F., DOCKERTY, M. B. AND COMFORT, M. W.—(1954) *Surg. Gynec. Obstet.*, **98**, 710.
SCHINDLER, R.—(1950) ‘ Gastroscopy.’ Chicago (University of Chicago Press).
SPRIGGS, E. I.—(1943) *Quart, J. Med.*, **12**, 1.
STEWART, M. J.—(1913) *J. Path. Bact.*, **18**, 127.—(1929) *Brit. med. J.*, ii, 567.
STOUT, A. P.—(1945) *N.Y. St. J. Med.*, **45**, 973.
WILLIS, R. A.—(1948) ‘ Pathology of Tumours.’ London (Butterworth).